# The crosstalk between circular RNAs and the tumor microenvironment in cancer metastasis

**DOI:** 10.1186/s12935-020-01532-0

**Published:** 2020-09-11

**Authors:** Ying Shao, Bingjian Lu

**Affiliations:** Department of Surgical Pathology, Women’s Hospital, School of Medicine, Zhejiang University, Hangzhou, Zhejiang China

**Keywords:** Metastasis, Circular RNA, Tumor microenvironment, Invasion, Cancer

## Abstract

**Background:**

Carcinomas are highly heterogeneous with regard to various cancer cells within a tumor microenvironment (TME), which is composed of stromal cells, blood vessels, immunocytes, and modified extracellular matrix.

**Focus of the study:**

Circular RNAs (circRNAs) are non-coding RNAs that are expressed in cancer and stromal cells. They are closely associated with cancer metastasis as their expression in tumor cells directs the latter to migrate to different organs. circRNAs packaged in exosomes might be involved in this process. This is particularly important as the TME acts in tandem with cancer cells to enhance their proliferation and metastatic capability. In this review, we focus on recent studies on the crosstalk between circRNAs and the TME during cancer metastasis.

**Conclusion:**

We particularly emphasize the roles of the interaction between circRNAs and the TME in anoikis resistance, vessel co-option, and local circRNA expression in directing homing of exosome.

## Background

The “seed-soil” description proposed by Stephen Paget in 1889 [1] is the classical theory of cancer metastasis. Cancer cells move from the primary neoplasm to a target distant location. The host environment, described as the “soil”, is remodeled to facilitate tumor metastasis, and the “seed” represents the cancer cells colonizing a new site suitable for tumor growth and progression. The cancer invasion-metastasis cascade consists of a series of discrete biological processes. Initially, cancer cells invade the surrounding tissue and enter the circulation, followed by their extravasation into a new site where they evade immune surveillance and anoikis to develop into metastatic tumors [2]. Various studies have considered the influence of the tumor microenvironment (TME) on cancer metastasis. The TME is composed of stromal cells, immune cells, vasculature, and extracellular matrix (ECM) [3]. Pervasive stromal reprogramming and ECM remodeling in the TME play vital roles in cancer metastasis [4].

Circular RNAs (circRNAs) are a novel type of non-coding RNAs that form covalently closed loop structures without 5′-caps or 3′-polyadenylated tails. They are ubiquitous and play critical roles in carcinogenesis. Cumulative evidence has shown that circRNAs are closely associated with cancer metastasis [5–7]. The interaction between circRNAs and the TME has significant clinical relevance to cancer therapy [8, 9]. Therefore, in the current review, we focus on the crosstalk between circRNAs and the TME in cancer metastasis.

## Biogenesis and functions of circRNAs

CircRNAs are produced by back splicing, which is different from the classical splicing of linear RNAs. Exon skipping and intron pairing narrow the distance between splice sites and facilitate the back-splicing of pre-mRNA [10]. This results in circRNAs lacking 3′ and 5′ ends and being more stable. CircRNAs are mainly classified into four types: exon circRNA (ecRNA), circular intron RNA (ciRNA), exon-intron circRNA (ElciRNA) and tRNA intronic circRNA (tricRNA) [11]. RNA-binding proteins (RBPs) and the spliceosome can regulate the circRNA biogenesis, whereas some of them are regulated by the TME [12, 13]. For example, the expression of circARSP91 in human hepatocellular carcinoma (HCC) is suppressed via upregulation of ADAR1 p110 caused by the androgen receptor (AR) [12]. HNRNPC is another RBP involved in circRNA biogenesis during hypoxic stress in cervical, breast, and lung cancer cell lines [13]. The function of circRNAs depends on their cellular localization; in the cytoplasm, they can be translated into peptides and sponge miRNAs, while in the nucleus, they may bind RBPs to influence transcription and modulate targeted pathways [14].

## The TME regulates tumor metastasis

The TME is composed of stromal cells, ECM components, immune cells, vasculature, other cell types, and various signaling entities, including exosomes [15]. The crosstalk between cancer cells and other TME components drives tumor progression and metastasis [16]. Cell types within the TME with specific roles in tumor metastasis include fibroblasts, endothelial cells, immune cells, adipocytes, and neuroendocrine (NE) cells. Apart from the ECM, other acellular components, such as extracellular vesicles (EVs) and cytokines released by tumor and non-tumor cells have also been identified [17–19]. Tumor cells communicate with stromal cells to promote tumor metastasis as part of the invasion-metastasis cascade. Cancer-associated fibroblasts (CAFs) represent the largest proportion of stromal cells in the TME; interestingly, their origin remains unclear. CAFs are activated by growth factors and cytokine including transforming growth factor-β (TGF-β) and fibroblast growth factor (FGF), which are released by tumor, stromal, and immune cells. Upon activation, CAFs secrete growth factors to support tumor progression, such as vascular endothelial growth factor (VEGF) that induces vascular permeability and angiogenesis. The YAP transcription factor is required for the CAF-mediated remodeling of the ECM to promote tumor progression [16]. In addition, CAFs can drive and direct cancer cell migration via fibronectin alignment [20]. Tumor-associated macrophages (TAMs), another important cell type within the TME, can be recruited by factors released from tumor cells and contribute to tumor progression during the metastatic cascade [21]. Moreover, they receive tumor-cell derived signals in the form of exosomes to promote metastasis [22]. Endothelial cells in the TME release adhesion molecules and chemokines to promote tumor progression, and can be stimulated by multiple factors secreted by tumor cells to promote angiogenesis, such as the basic fibroblast growth factor (bFGF) and VEGF [23, 24]. With regard to the acellular TME components, the ECM builds a three-dimensional network composed of collagen, fibronectin, elastin, proteoglycans, laminins, and other glycoproteins [18]. These components bind to cell surface receptors and transmit signals to cells from the ECM, thereby inducing tumor migration, differentiation, metabolic alterations, and other changes in cellular behavior [25]. A change in cell adhesion to the ECM is an important step in metastasis. Notably, the matrix metalloproteinase (MMP) family members are the main proteolytic enzymes to degrade the ECM component and promote tumor cell migration via the basement membrane (BM). MMPs can be produced by stromal and tumor cells. Specifically, MMP13 expression is mainly observed in CAFs, but this enzyme may also be synthesized by other cells to promote invasiveness and angiogenesis [26]. MMP10 supports lung cancer development and metastasis [27]. Recent studies have shown that primary tumor sites may send signals to distant homing locations via primary tumor-derived exosomes. The delivery of such signals may contribute to organ tropism, immune evasion, metastasis, and can be used to predict patient outcomes [16].

## **The crosstalk between circRNAs and the TME in local invasion and migration**

Invasion is the initial step of metastasis. During this process, tumor cells alleviate their adherence to neighboring cells and the ECM, followed by the degradation of surrounding tissues and the acquisition of enhanced motility to navigate through tissues [2].

### Loosened cell-cell adhesion

Cell-cell adhesions are mediated by cadherins, which, through their extracellular domains, bind catenin and actin cytoskeletal proteins intracellularly. Cadherin expression in cells changes from E-cadherin to N-cadherin during invasion. The former promotes tumor cell-cell adherence, while the latter is expressed on mesenchymal cells and induces tumor cells to bind to the stroma to promote invasion [2]. Macrophages are the main stromal cells involved in the circRNA crosstalk to regulate cadherin expression in tumor cells.

**Fig. 1:**
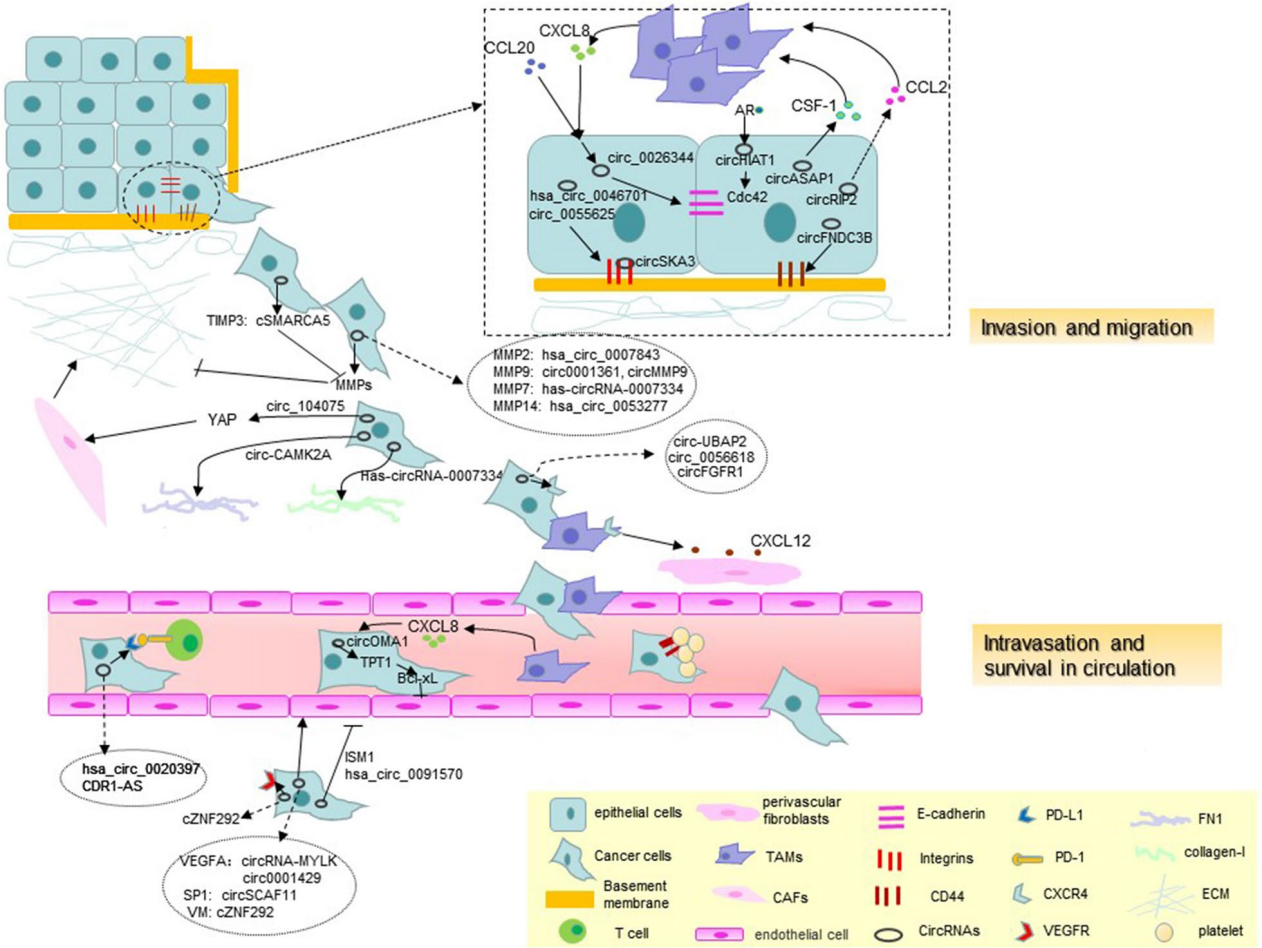
The crosstalk between circRNAs and TME in cancer invasion and intravasation. In invasion and migration, circASAP1 and circRIP2 from tumor cells contribute to tumor-associated macrophage (TAM) recruitment through CSF-1 and CCL2, respectively, while CXCL8 from TAMs can affect circ_0026344 expression in tumor tissue when interacting with CCL20 to influence cell-cell adhesions. Many circRNAs target MMPs and TIMPs to degrade the basement membrane and interstitial matrix. Circ_005625, circFNDC3B, and other circRNAs can modulate integrins, CD44, collagen-1, and FN1 to influence the interaction between tumor cells and ECM. CircRNAs can contribute to cancer-associated fibroblast (CAF)-mediated remodeling of the ECM by targeting the YAP transcription factor. During intravasation, circRNAs regulate CXCR4 on TAMs, which are attracted by CXCL12 from perivascular fibroblasts to migrate toward the blood vessel. CircRNAs from cancer tissues can regulate the immune escape by targeting PD-L1 and influence angiogenesis by modulating VEGFA, VEGFR, SP1, and ISM1 expression. CircRNAs are involved in cancer cell survival in the circulation by targeting CD44 and anoikis-related protein Bcl-xL to resist cell arrest and anoikis

Macrophages are recruited to the TME by tumor cells via the secretion of chemokines or cytokines. Once in the TME, they are polarized to a tumor-associated macrophage (TAM) phenotype [28]. The infiltration of TAMs in the TME leads to the migration, invasion, and enhanced pro-angiogenic capacity of bladder cancer cells through CXCL8 secretion, which in turn regulates cadherin expression to accelerate cancer invasion [29]. Low expression of circ_0026344 was detected after CCL20 and CXCL8 synergized treatment in colorectal cancer (CRC) cell lines. This circRNA sponged miR-183 to downregulate E-cadherin and upregulate N-cadherin and vimentin expression via the PI3K/AKT/ERK pathway [30]. These observations suggest that chemokines from immune cells, particularly TAMs in the TME, influence cancer cell gene expression during tumor metastasis (Fig. 1).

Direct contact through TAM recruitment can mediate cell-cell adhesion. TAMs can promote CRC migration, invasion, and circulating tumor cell (CTC)-mediated metastasis via the JAK2/STAT3/miR-506-3p/FoxQ1 axis, enhancing macrophage recruitment through the production of CCL2 by tumor cells [31]. These results demonstrate that TAMs promote CRC metastasis through a positive feedback loop. Recently, Su et al. [32] showed that high circRIP2 (has_circ_0005777) levels were associated with a favorable outcome in patients with bladder cancer. However, circRIP2 overexpression promoted bladder cancer cell proliferation and metastasis in vitro and promoted bladder cancer growth in nude mice. Further investigation suggested that circRIP2-accelerated bladder cancer progression occurred via the miR-1305/TGF-β2/smad3 pathway with downregulation of E-cadherin and upregulation of N-cadherin and vimentin [32]. Interestingly, there was an inconsistency between the clinical association and biological functionality of circRIP2 expression in bladder cancer. RNA-sequencing of circRIP2-overexpressing cells revealed that with the exception of TGF-β signaling, chemokines (CCL2, CCL3, CXCL5, CXCL17, and CXCL20) and cytokines (IL-6, IL-13, and IL-17) were also differentially expressed [32]. Accordingly, TAMs may be recruited via CCL2 to mediate bladder cancer metastasis through indirect crosstalk with tumor circRNAs (Fig. 1).

Additionally, circASAP1 (hsa_circ_0085616) overexpression was associated with pulmonary metastasis in HCC patients. Further, this circRNA promoted cancer cell proliferation and invasion via miR-326/miR-532-5p-MAPK1 signaling in addition to TAM-mediated infiltration via the miR-326/miR-532-5p-CSF-1 pathway [33]. In summary, circRNAs may contribute to a novel mechanism of TAM recruitment leading to tumor invasion and metastasis (Fig. 1) (Table 1).

**Table 1:** The crosstalk between circRNAs and the TME in cancer metastasis

Category	circRNA	Expression in cancers	TME components	Mechanisms of action	References (#)
Local invasion and migration					
	circ_0026344	CRC↓	TAMs	circ_0026344/miR-183	[30]
	circRIP2	BC↑	TAMs	circRIP2/miR-1305/TGF-β2-smad3	[32]
	circASAP1	HCC↑	TAMs	CircASAP1/miR-326/miR-532-5p/MAPK1-CSF-1	[33]
	hsa_circ_0007843	CC↑	ECM	hsa_circ_0007843/miR-518c-5p/MMP2	[38]
	hsa_circ_0001361	BC↑	ECM	hsa_circ_0001361/miR-491-5p/MMP9	[39]
	CircMMP9	OSCC↑	ECM	CircMMP9/ miR-149/ MMP9	[40]
	has-circRNA-0007334	PDAC↑	ECM	Has-circRNA-0007334/hsa-miR-144-3p/MMP7	[43]
	cSMARCA5	HCC↓	ECM	cSMARCA5/miR-17-3p/miR-181b-5p/TIMP3	[46]
	hsa_circ_0053277	CRC↑	ECM	Hsa_circ_0053277/miR-2467-3p/MMP14	[48]
	circ_0055625	CC↑	ECM	circ_0055625/miR-106b-5p (miR-106b)/ITGB8	[54]
	hsa_circ_0046701	Glioma↑	ECM	hsa_circ_0046701/miR-142-3p/ ITGB8	[55]
	circFNDC3B	GC↑	ECM	CircFNDC3B/IGF2BP3/CD44 mRNA	[58]
	has-circRNA-0007334	PDAC↑	ECM	Has-circRNA-0007334/has-miR-577/COL1A1	[43]
	circ-CAMK2A	LUAD↑	ECM	circ-CAMK2A/miR-615-5p /FN1	[61]
	circSKA3	Breast cancer↑	ECM	circSKA3 Tks5-integrinβ1	[63]
	circRNA_104075	HCC↑	CAFs	circ_104075 / miR-582-3p/YAP	[65]
	circHIAT1	CCRCC↓	AR	circHIAT1/miR-195-5p/29a-3p/Cdc42	[67]
Intravasation					
	circ-UBAP2	PAAD↑	TAMs	circ-UBAP2/hsa-miR-494/CXCR4 and ZEB1	[73]
	circ_0056618	GC↑	TAMs	circ_0056618/miR-206/CXCR4	[74]
	circFGFR1	NSCLC↑	TAMs	circFGFR1/miR-381-3p/CXCR4	[75]
	hsa_circ_0020397	CRC↑	Immunocytes	hsa_circ_0020397/miR-138/TERT and PD-L1	[82]
	CDR1-AS	CC↑	Immunocytes	Unknown	[83]
	circRNA-MYLK	BC↑	Angiogenesis	circRNA-MYLK/miR-29a/VEGFA	[88]
	circ0001429	BC↑	Angiogenesis	circ0001429/miR-205-5p/VEGFA	[89]
	circSCAF11	Glioma↑	Angiogenesis	circSCAF11/miR-42 /SP1/VEGFA	[90]
	cZNF292	Glioma cells, unknown	Angiogenesis	Unknown	[92]
	hsa_circ_0091570	HCC↓	Angiogenesis	hsa_circ_0091570/miR-1307/ISM1	[94]
	cZNF292	HCC↑(hypoxia-responsive)	Angiogenesis	ccZNF292/SOX9/VM	[95]
Survival in circulation					
	circRNA-seq	Human platelets, unknown	Platelet-tumor cell clusters		[101]
	circ_0026344	CRC↓	Anoikis resistance	circ_0026344/miR-183	[30]
	circOMA1	NFPA↑	Anoikis resistance	circOMA1/miR-145-5p/TPTI/Bcl-xL	[105]
Exosomes on homing					
	circPUM1	EOC↑		CircPUM1/miR-6753-5p/MMP2	[106]
	circ-IARS	Pancreatic cancer↑		circ-IARS/miR-122 /RhoA/F-actin and ZO-1	[107]
	hsa_circ_0137439	Bladder cancer urine↑		hsa_circ_0137439/miR-142-5p/MTDH	[110]
	hsa_circRNA_0056616	LUAD↑		Unknown	[113]

↑ upregulation; ↓ downregulation; ECM: extracellular matrix; CRC: colorectal cancer; BC: bladder cancer; HCC: hepatocellular carcinoma; CC: colon cancer; OSCC: oral squamous cell carcinoma; PDAC: pancreatic ductal adenocarcinoma; TIMPs: tissue inhibitors of metalloproteinases; ITGB8: integrin β8; GC: gastric cancer; COL1A1: collagen type I α 1; FN1: fibronectin 1; LUAD: lung adenocarcinoma; CAFs: cancer-associated fibroblasts; CCRCC: clear cell renal cell carcinoma; AR: androgen receptor; PAAD: pancreatic adenocarcinoma; NSCLC: non-small cell lung cancer; TERT: telomerase reverse transcriptase; ISM: Isthmin; VM: vasculogenic mimicry; SOX9: SRY (sex determining region Y)-box 9; TPT1: translationally controlled tumor protein; NFPAs: non-functioning pituitary adenomas; EOC: epithelial ovarian cancer; MTDH: metadherin

### Degradation of the basement membrane and interstitial connective tissue

During invasion, altered tumor cells adherence to the ECM and neighboring cells is followed by the recruitment of proteases to degrade the BM and interstitial connective tissue, providing a pathway for invasion [34]. Tumor cells secrete proteolytic enzymes and induce stromal cells to do the same. MMPs are the main proteases involved in the degradation of the BM and interstitial matrix [35]. Among the MMP family, MMP2 and MMP9 are the main proteases involved in the breakdown of the BM [36, 37]. Hsa_circ_0007843 was highly expressed in colon cancer tissues and cells. It sponged miR-518c-5p to upregulate MMP2 expression and further affected the invasive capability of colon cancer cells [38]. Hsa_circ_0001361 (circ0001361) was highly expressed in bladder cancer tissues and cell lines, and its expression correlated with pathologic grade and invasion of the muscular wall. Mechanistically, circ0001361 directly interacts with miR-491-5p to upregulate MMP9 expression, promoting bladder cancer cell invasion and metastasis both in vitro and in vivo [39]. CircRNAs can regulate MMP9 mRNA stability to promote tumor cell invasion. The circMMP9 (hsa_circ_0001162) was strikingly upregulated in oral squamous cell carcinoma (OSCC) and significantly correlated with MMP9 expression, lymph node metastasis, and an advanced TNM stage. Mechanistically, circMMP9 interacted simultaneously with AUF1 and miR-149 to block the inhibitory effect of AUFI and miR-149 on the MMP9 3’-untranslated region, contributing to enhanced MMP9 mRNA stability and the subsequent promotion of metastasis [40].

MMP7, known as a prometastatic factor, is a type of matrilysin, which cleaves various protein components of the ECM, including collagen, entactin, fibronectin, and laminin among others [41, 42]. Has-circRNA-0007334 was upregulated and sponged hsa-miR-144-3p to enhance the expression and function of MMP7 and promote pancreatic ductal adenocarcinoma (PDAC) invasion [43].

Tissue inhibitors of metalloproteinases (TIMPs) are widely distributed in the human body [44]. The balance between the MMPs and TIMPs is a key factor for the maintenance of a normal configuration of the ECM and BM, preventing tumor cell invasion and migration [45]. In HCC, the downregulation of circSMARCA5 (hsa_circ_0001445) promoted the expression of TIMP3 by sponging miR-17-3p and miR-181b-5p [46]. MMP14 was reported to degrade the ECM by increasing the secretion of pro-MMP2, pro-MMP9, and interacting with TIMP2 [47]. Furthermore, hsa_circ_0053277 was remarkably upregulated in CRC tissues and cells, which contributed to tumor cell invasion by sponging miR-2467‐3p to target MMP14 [48] (Fig. 1) (Table 1).

### Altered tumor cell adhesion to ECM proteins

Integrins are the main mechanoreceptors on tumor cells that bind ECM proteins to mediate bidirectional signaling across cell membranes. They couple diverse extracellular ligands to the cytoskeleton [49] and play a key role in metastasis-linked ECM modifications [50]. Integrins consist of two transmembrane glycoproteins, α and β [51]. While α subunits bind to ECM proteins to form large multiprotein complexes known as focal adhesions, β subunits are responsible for interacting with the actomyosin cytoskeleton to affect cellular mobility via the focal adhesion kinase signaling pathway [52, 53]. Integrins play roles in tumor metastasis and regulate CTC survival in the circulation by easing anchorage-independent death. CircRNAs are involved in integrin expression in CC and glioma. Circ_0055625 was upregulated in CC to promote tumor growth and invasion via circ_0055625-miR-106b‐5p-integrin β8 (ITGB8) axis. ITGB8 is a member of the integrin family, which binds through its α subunit and plays major roles in regulating cell invasion [54]. ITGB8 was targeted through increased expression of hsa_circ_0046701 sponging miR-142-3p to promote glioma progression [55].

CD44 is another tumor cell receptor for ECM proteins, more specifically, a highly polymorphic receptor for hyaluronan (HA), part of the immunoglobulin receptor superfamily, and a surface proteoglycan. CD44 proteins comprise a major class of single-pass transmembrane glycoproteins and connect the extracellular matrix to the cytoskeleton to induce downstream signaling for the promotion of tumor invasion [56]. The interaction of HA and CD44 can contribute to cell-cell loosening with the upregulation of ZEB1 and downregulation of E-cadherin [57]. The direct link between circRNAs and CD44 was initially reported in gastric cancer (GC) cells. CircFNDC3B (hsa_circ_0006156) was associated with the degree of differentiation of GC cells and promoted their migration and invasion via a novel mechanism, namely the formation of a ternary complex of cirFNDC3B-IGF2BP3-CD44 mRNA. Silencing of circFNDC3B increased E-cadherin levels and reduced N-cadherin levels. Insulin-like growth factor 2 binding protein 3 (IGF2BP3), a RNA binding protein, directly targets CD44 mRNA to increase its stability [58].

Moreover, circRNA expression in cancer tissue directly targets ECM proteins. Collagen type 1, as the major ECM protein of all fibrous stroma, provides structural and biochemical cues to cells within the stroma. The accumulation of type 1 collagen is a notable property of “cancerized” stroma. Collagen crosslinking increases stroma stiffness to promote tumor progression [59]. Collagen type 1 α 1 (COL1A1), which encodes the pro-α 1 chains of type 1 collagen, is targeted by circRNA. Has-circRNA-0007334 sponged has-miR-577 to upregulate COL1A1 expression and to promote PDAC progression [43]. Fibronectin 1 (FN1) is a glycoprotein in the ECM, which can mediate the metastasis of various cancers by activating MMP2 and MMP9 expression [60]. Circ-CAMK2A (hsa_circ_0128332) overexpression was considerably associated with lymph node metastasis, distant metastasis, and poor clinical outcomes in lung adenocarcinoma (LUAD). It became evident that this circRNA sponged miR-615-5p to target FN1 expression, thereby, contributing to the metastasis of LUAD [61].

Besides the chemical properties, integrins are also important components of invadopodia, the invasive structures which sense the physical properties of the ECM and regulate tumor invasion. The highest filopodia density occurred when cancer cells invaded through a gel composed of BM constituents as revealed in a study on filopodia formation in breast cancer cells. It has been suggested that cancer cell-intrinsic mechanisms assess the surrounding matrix potential via integrin receptor signaling resulting in invadopodia formation [62]. Recently, it has been shown that circRNA directly binds to integrins and regulates invadopodia formation to degrade the ECM. In breast cancer, circSKA3 directly bound with Tks5 and integrin β1 to form a complex with tumor-derived invadopodia, thus promoting tumor invasion [63]. The YAP transcription factor is required for CAF-mediated ECM remodeling. It was also found to be regulated by circRNAs [64]. CircRNA_104075 (circ_104075) expression increased in cancer tissues and sera from HCC patients. HNF4a bound to the circ_104075 promoter region to upregulate expression of circ_104075. As a ceRNA, circ_104075 absorbed miR-582-3p to upregulate YAP expression via an N6-methyladenosine (m6A) motif [65]. CircRNA-associated YAP expression in HCC may induce CAFs to remodel the ECM to further promote tumor progression. This crosstalk may represent a new mechanism in CAF-mediated ECM remodeling (Fig. 1) (Table 1).

### Promoting migration

Migration is the final step of invasion and involves impingement on the actin cytoskeleton. Integrins transmit signals from ECM components to the actin cytoskeleton inside of the cell. Some of the key players linking integrins to the actin cytoskeleton are small Rho family members, such as Rac, Cdc42, and RhoA [66]. They are the functional regulators of the actomyosin contractile machinery and promote cell migration by enhancing cell body contraction. Cdc42 is responsible for inducing cell polarity and directly determining the persistence of motion [52]. A study of the antigen receptor (AR) in clear cell renal cell carcinoma (CCRCC) revealed that AR-mediated suppression of circHIAT1 (hsa_circ_001013) could enhance CCRCC cell invasion and migration via miR-195-5p/29a-3p/29c-3p/Cdc42 signaling. This could explain why men with CCRCC have a higher survival rate than women [67]. Importantly, this study suggested that the hormonal microenvironment might influence the mobility of cancer cells (Fig. 1) (Table 1).

## The crosstalk between circRNAs and the TME in intravasation

Intravasation refers to cancer cells disseminating into the lumen of vasculature in order to travel to distant sites for metastasis. The endothelium poses a barrier for tumor cell intravasation [68]. TAMs are the main stromal cells to localize to blood vessels and help tumor cell intravasation [69]. Two TAM subsets are involved in intravasation: migratory macrophages are responsible for guiding cancer cells toward blood vessels, while sessile perivascular macrophages assist them into vessels. Monocytes are initially recruited via CCR2 signaling. Cancer cells induce TGF-β-dependent upregulation of C-X-C motif chemokine receptor 4 (CXCR4) on monocytes. Monocytes are then attracted by perivascular fibroblasts, which express CXCL12 around blood vessels, and bring along motile cancer cells [70]. Subsequently, migratory TAMs differentiate into perivascular macrophages to promote vascular leakiness and intravasation via Tie2-mediated attachment to endothelial cells [71]. Accordingly, CXCR4-expressing macrophages are called “transporters”, which carry tumor cells toward the blood vessel and present them to perivascular macrophages. The CXCR4 expression level was significantly higher in non-small cell lung cancer (NSCLC) tissues from the patients with lymph node metastasis than in those without metastasis. Interestingly, high CXCR4 expression in tumor tissue paralleled its expression in TAMs [72]. Recently, a study on circRNAs targeting CXCR4 expression has revealed the association between tumor cells and TAM infiltration. In pancreatic adenocarcinoma (PAAD), circ-UBAP2-hsa-miR-494 potentially targets CXCR4 and ZEB1 transcripts. Expression of CXCR4 and ZEB1 regulated PAAD by modulating the infiltration and function of immune cells, including TAMs [73]. CXCR4 is the chemokine receptor in tumors and chemokine CXCL12 is the specific ligand that interacts with it. As per the study described above, the CXCR4/CXCL12 interaction is important for migration to the blood. Tumor cells expressing CXCR4 may be attracted toward blood vessels by perivascular fibroblasts that express CXCL12 via synergizing with CXCR4-expressing TAMs. Notably, circRNAs targeting CXCR4 in tumor tissue represent a new insight in TAM-associated tumor invasion. Circ_0056618 was overexpressed in GC tumor tissues and cells. Inhibition of circ_0056618 suppressed tumor cell proliferation and metastasis by preventing sponging of miR-206 and the subsequent targeting of CXCR4 [74]. Furthermore, in NSCLC, circFGFR1 (hsa_circ_0084003) expression increased in cancer tissues and promoted cell migration, invasion, and immune evasion by interacting with miR-381-3p to target CXCR4 [75]. CircRNA-modulated CXCR4 expression may represent an important regulatory mechanism for TAM-mediated intravasation (Fig. 1) (Table 1).

## Immune escape

During invasion and intravasation, tumor cells should avoid immune recognition in order to survive before extravasating. Some reviews have elaborately summarized the association among circRNAs TME immune cells and the immune escape [8, 9]. Herein, we focus on the crosstalk between circRNAs and immune cell function in relation to invasion. Tumor immunity mainly involves in T cells and dendritic cells (DCs), and exploits immune checkpoints, which are normally critical for the prevention of autoimmunity, such as cytotoxic T lymphocyte-associated antigen 4 (CTLA4, CD152) and programmed cell death protein 1 (PD-1) [76]. Cytotoxic CD8+ and CD4+ Th1 T cells are the major antitumor immune effector cells, whose antitumor functions are amplified by immune checkpoint blockade antibodies such as CTLA4 inhibitors and PD-1/PD-L1 inhibitors [77]. PD-1 belongs to the B7/CD28 family and is expressed on activated T cells [78]. PD-1 binds to PD-L1 and PD-L2 to decrease T cell proliferation and induce apoptosis [17, 79]. In lung adenocarcinoma (LADC) and lung squamous cell carcinoma (LSCC), epithelial gene markers showed a general negative correlation with the expression of immune checkpoint genes, while the opposite was seen for mesenchymal gene markers [80]. In melanoma, subsets of tumors inherently resistant to immunotherapy demonstrated innate anti-PD-1 resistance (IPRES) [81]. Immune checkpoints seem to be the main factors leading to immune suppression-associated cell-cell adhesion. As previously reported, circRNAs directly influence PD-L1 expression. In CRC, an upregulation of hsa_circ_0020397 inhibited miR-138 activity by targeting telomerase reverse transcriptase (TERT) and PD-L1, and, consequently, regulated CRC cell viability, apoptosis, and invasion [82]. CDR1-AS can regulate cell surface PD-L1 protein levels independently of microRNA to promote the malignant behavior of CC cell lines [83] (Fig. 1) (Table 1).

## Angiogenesis

Angiogenesis plays an important role in the process of intravasation. It is a crucial adaptive event, allowing for tumor growth beyond 1–2 mm^3^ [84]. Blood supply is required for the growth of metastases by providing oxygen, growth factors, nutrients, and metabolites. VEGF is the main inducer of angiogenesis and also induces vascular permeability [2]. Some circRNAs directly target VEGF expression. VEGFA (a member of the VEGF family) and the VEGFA receptor VEGFR-1/2 play key regulatory roles in tumor endothelial tube formation [85–87]. In bladder cancer, circRNA-MYLK binds to miR-29a to target VEGFA, which normally activates its receptor VEGFR2 and the downstream Ras/ERK signaling pathway, contributing to tube formation and cytoskeletal rearrangement [88]. Circ0001429 interacts with miR-205-5p to regulate VEGFA expression, accelerating cell propagation, migration, and invasiveness in bladder cancer [89]. In glioma, circSCAF11 (hsa_circ_0098619) overexpression can sponge miR-42 to positively regulate the expression of transcription factor SP1, which can bind to the promoter region of VEGFA to enhance its expression [90]. Furthermore, VEGFR-positive tumor cells create an autocrine loop that also affects non-angiogenic aspects of tumor cell progression [91]. CircRNAs targeting VEGFR have been reported as upstream regulators of the autocrine loop. circZNF292 was confirmed to induce tube formation through its association with genes such as VEGFR-1/2 and p-VEGFR-1/2 in glioma [92].

Isthmin (ISM) is another endogenous angiogenesis inhibitor that has the capacity to block angiogenesis by VEGF and bFGF. It mitigates VEGF-stimulated endothelial cell proliferation without affecting endothelial cell migration and binds to integrins on the endothelial surface to support adhesion [93]. In HCC, hsa_circ_0091570 was downregulated and functioned as a sponge of miR-1307 to regulate the expression of ISM1 [94]. The tumor blood supply is influenced by vasculogenic mimicry (VM) that has the features of intratumoral channels lined by tumor cells and lacking inner endothelial cell lining. Notably, erythrocytes and plasma can flow in these vessel-like structures. VMs are connected with host vessels for blood supply [4]. In HCC, hypoxia-induced circZNF292 can affect the VM through SRY (sex determining region Y)-box 9 (SOX9) nuclear translocation, a downstream transcription factor in the Wnt/β-catenin pathway, to promote tumor progression [95] (Fig. 1) (Table 1).

## **The crosstalk between circRNAs and TME for tumor cell survival in the circulation**

After intravasation, tumor cells stuggle to survive through the circulatory system and eventually undergo arrest in the vasculature, followed by extravasation to form distant metastases. It is estimated that only 0.01% of cells that intravasate into circulation can form detectable metastases [96]. Interaction with platelets and anoikis resistance both play important roles during this process.

### Interaction with platelets

Selectins, a family of transmembrane cell adhesion molecules, are expressed by platelets, endothelial cells, and leukocytes. Platelets and leukocytes can use P-selectin to support the initial transient tumor cell interaction with the endothelium [97]. CD44 expressed on tumor cells binds to platelet P-selectin and interacts with fibrin to establish firm adhesion between platelets and tumor cells. Thus, tumor cells cooperate with platelets and leukocytes to form hetero-aggregates that travel along the areas of activated endothelium in the circulation in a P-selectin-dependent manner [98, 99]. CD44 could be described as a “bridge” that links tumor cells and platelets to help tumor cell arrest in circulation. As described above, the presence of CD44-associated circRNAs in tumor cells may provide new clues regarding the tumor cell-platelet interaction (Fig. 1).

After selectin-dependent rolling along the endothelium, integrins mediate the firm arrest of CTCs to the vessel wall. Platelet integrin αIIbβ3 is involved in this process, as it cooperates with tumor cell receptors to mediate tumor cell-platelet cohesion and arrest to the vessel wall [100]. During this process, when platelets are activated, platelet-tumor cell clusters ensure immune evasion and contribute to tumor progression by releasing platelet granules and microparticles. CircRNAs can be delivered in vesicles, and be transported to tumor cells. A circRNA-seq analysis of isolated extracellular vesicles (EVs) from purified and in vitro activated human platelets has showed that circRNAs are specifically abundant, packaged, and released from both types of vesicles, including 32 circRNAs from microvesicles and 35 from exosomes. These findings suggest a specific sorting mechanism of circRNAs in EVs [101]. It was documented that platelet-derived TGF-β and direct platelet-tumor cell contacts synergistically activated the TGF-β/smad and NF-kB pathways to enhance metastasis in vivo. After co-incubation of tumor cells with purified platelets, increased numbers of metastatic foci were observed in the lungs [102]. These results indicated that a transient interaction between tumor cells and platelets was sufficient to promote tumor cell metastasis. However, the exact interaction mechanism of how platelets deliver signals to tumor cells remains unclear to date. Platelet-derived circRNAs in microvesicles or exosomes might be involved in this process.

### Anoikis resistance

The loss of adhesion to ECM proteins leads to apoptosis in normal cells. This process is called anoikis, a specific type of programmed cell death. Anoikis resistance is a critical mechanism involved in cancer invasion and metastasis. A small number of CTCs in the blood can resist anoikis in the bloodstream through increasing the expression of CXCL8, but it is still unclear how CXCL8 and its receptors regulate anoikis resistance [103]. Recently, one study revealed that TAM infiltration in the TME elevates CXCL8, promoting bladder cancer cell migration and invasion via the secretion of MMP9, VEGF, and E-cadherin [29]. Further, TAMs carried tumor cells into the bloodstream. CCL20 and CXCL8 suppressed circ_0026344 expression to promote invasion in CRC cells [30]. TAMs secreting CXCL8 may promote anoikis resistance outside and inside the circulation by regulating circRNA expression in tumor cells and CTCs.

Moreover, during the process of CTCs avoiding anoikis to attach to the endothelium, CTC chemokine receptors CXCR4 and CCR7 bind to CXCL12 and CCL21 on endothelial cells to mediate tumor cell arrest and to resist anoikis by regulating the proapoptotic and antiapoptotic Bmf and Bcl-xL proteins [104]. A limited number of studies have investigated on circRNAs and anoikis-related protein Bcl-xL at present. In non-functioning pituitary adenomas (NFPAs), miR-145-5p strikingly decreased and negatively correlated with NFPA invasiveness. In addition, it had a positive correlation with apoptosis [105]. circOMA1 (hsa_circRNA_0002316) sponged miR-145-5p to abrogate its suppressive effect on invasion in NFPA. Translationally controlled tumor protein (TPT1) was found to be downstream of the circOMA1 /miR-145-5p axis. CircOMA1 could upregulate downstream factors of TPT1, Mcl-1, Bcl-xL, and downregulate Bax [105]. CircRNAs may be a part of a previously unknown axis implicated in invasion that connects chemokine receptors with anoikis resistance in the absence of the appropriate cell-ECM interaction (Fig. 1) (Table 1).

## Exosomal circRNAs affect tumor homing

CircRNAs derived from exosomes can influence MMP2 expression to promote distant metastasis. CircPUM1 (circ_0000043) was highly expressed in epithelial ovarian cancer (EOC) tissues and upregulated MMP2 expression by sponging miR-6753-5p. CircPUM1 was found in cancer cell-derived exosomes and could be transferred to act on the peritoneum, regulating peritoneal expression of MMP2 and, thus, promoting tumor dissemination [106].

The endothelial barrier is the main factor for intravasation and extravasation during metastasis. The endothelial-tumor cell interaction is a central determinant of where exactly metastatic tumor cells will exit circulation [16]. CircRNAs can directly influence the endothelial barrier via exosomes. Circ-IRAS packaged in exosomes was upregulated in pancreatic cancer and entered microvascular endothelial cells to promote liver metastasis, intravasation, and tumor node-metastasis stage [107]. Circ-IARS could sponge miR-122 in endothelial cells to upregulate RhoA expression. Activated RhoA increases F-actin expression and reduces the expression of tight junction protein Zonula occludens-1 (ZO-1), resulting in an enhanced inward contractile force of cells, compromised endothelial barrier function, and increased endothelial permeability [107].

Exosomal circRNAs from local tumor cells can signal to endothelial cells in distant organs in order to facilitate metastasis. Endothelial cells in different organs can express different surface proteins. A number of peptides selectively bind the endothelia of target organs [108]. In cancers, specific proteins expressed on endothelial cells in the target organ may be crucial in determining the site of tumor cell arrest. Metadherin (MTDH), a protein that is overexpressed in metastatic breast cancer, can bind to the vasculature of the lung, a common site of breast cancer metastasis [109]. CircRNAs targeting MTDH expression have been studied. In bladder cancer, hsa_circ_0137439 upregulation in urine supernatant was correlated with advanced tumor stage, high tumor grade, increased lymph node metastasis, and muscular wall invasion. Mechanistically, knocking down hsa_circ_0137439 contributed to the inhibition of cell proliferation and migration via the hsa_circ_0137439/miR-142-5p/MTDH axis [110]. Hsa_circ_0137439 may be found in cell-free urine supernatant as exosomal cargo or within other molecular vehicles. Tumor cells can use pre-existing tissue blood vessels to support themselves in the absence of angiogenic processes. This new form of tumor metastasis is called vessel co-option and has been reported in tumors of the brain, liver, lung, and lymph nodes [111]. Exosomal circRNAs targeting specific endothelial cell proteins might provide new insights into the metastatic process although the exact mechanisms remain unclear.

**Fig. 2:**
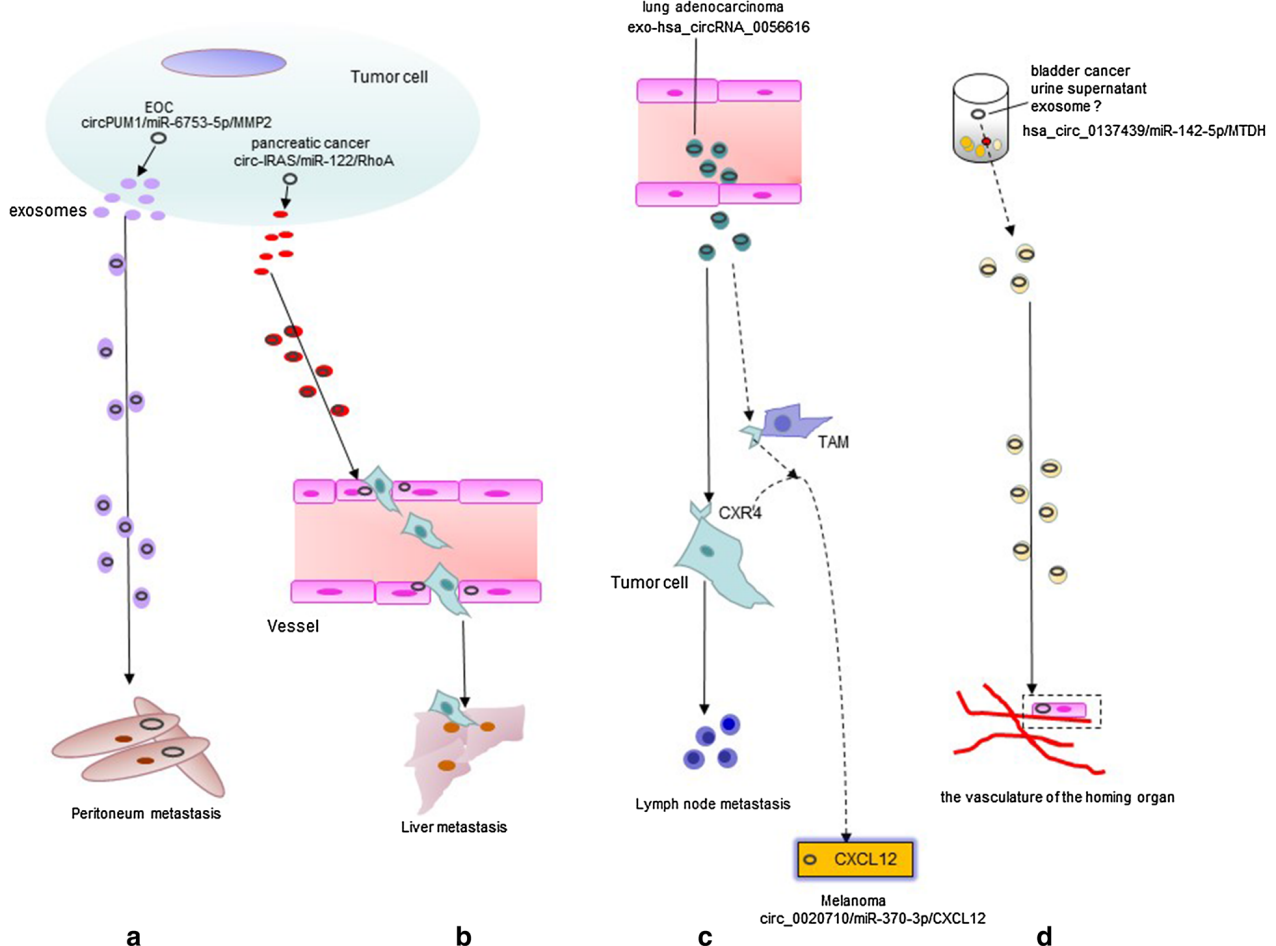
Exosomal circRNAs affect tumor homing. **a** Epithelial ovarian cancer (EOC)-derived exosomes containing circPUM1, which targets MMP2 transcripts, are transported to the peritoneum to regulate tumor metastasis. **b** In pancreatic cancer, exosomes containing circIRAS enter microvascular endothelial cells to compromise endothelial barrier function and to promote liver metastasis by targeting RhoA. **c** The CXCR4/CXCL12 axis is critical for cancer metastasis. In lung adenocarcinoma, exo-hsa_circRNA_0056616 participates in lymph node metastasis by targeting CXCR4 and affecting TAM infiltration. CXCR4 expression on TAMs or tumor cells drives the spread to the organs expressing CXCL12, which has been identified as regulated by circ_0020710 in melanoma. **d** In bladder cancer urine supernatant, hsa_circ_0137439 in exosomes or other vehicles targets metadherin (MTDH), which can bind to the vasculature of distant organs to promote metastasis (The solid arrow indicates verified observations, while the dotted arrow indicates hypothesis)

TAMs are the main stromal cells in the TME and may therefore contribute to tumor cell homing. CXCR4 was frequently expressed on breast cancer cells and guided tumor cell spread to the bone, lung, and regional lymph nodes expressing high levels of its ligand CXCL12 [112]. The CXCR4/CXCL12 interaction is central to tumor cell homing to distant organs. However, the underlying mechanism is not very clear. Recently, TAMs have been found to play a dominant role in mediating CXCR4/CXCL12-induced liver metastasis of CRC via exosomal miRNAs. miR-25-3p, miR-130b-3p, and miR-425-5p were upregulated in CRC cells and could be transferred to macrophages via exosomes after activation of the CXCR4/CXCL12 axis. Macrophages would uptake these miRNAs and be transformed into TAMs via PI3K signaling, thereafter promoting metastasis by enhancing the epithelial-mesenchymal transition and secreting VEGF. These miRNAs were also examined in serum exosomes and correlated with CRC progression and metastasis [22]. Thus, it can be reckoned that local tumor cell homing may be mediated by exosomes, which can facilitate the crosstalk with other cells within the TME. LUAD patients with lymph node metastasis had higher plasma levels of exo-hsa_circRNA_0056616 and CXCR4 expression than those without [113]. CircRNAs can build up a ceRNA network with miRNAs. CXCR4-related exo-circRNAs were involved in TAM-mediated metastasis toward organs with high expression of CXCL12. Elevated circ_0020710 was observed in melanoma tissues. It promoted cell proliferation, migration, and invasion in vitro and tumor growth in vivo via the miR-370-3p/CXCL12 axis [114] (Table 1). The roles of circRNAs in CXCR4/CXCL12-mediated distant metastasis are depicted in Fig. 2.

## Conclusion and future perspectives

In summary, we have reviewed multiple interactions between the TME and circRNAs involved in the metastatic process. These interactions form crucial regulatory networks that can be useful for predicting tumor progression. With the discovery of increasing numbers of circRNAs, the interaction between circRNAs and the TME has become a hot topic within molecular biology and oncology. A better understanding of this reciprocal cooperation is mandatory to identify important prognostic and predictive biomarkers for the development of novel cancer therapies. For example, one study found that primary tumor cells surrounded by platelets were localized to sites in which the epithelial-mesenchymal transition occurred based on molecular and morphological changes of HER2-negative breast cancer. Moreover, primary tumor cells associated with platelets exhibited chemoresistance to common anti-cancer drugs [115]. Understanding the mechanism through which platelet-associated exo-circRNAs contribute to tumor cell survival in circulation and extravasation to distant sites may provide important new insights. As an important cell type present within the TME, TAMs are central for the promotion of metastasis. The CXCR4/CXCL12 axis may play an important role in directing distant homing. As previously described, homing-associated circRNAs have been identified; however, the specific mechanism of TAM contributiing to tumor cell migration to vessels and survival in the circulation remains unclear. Personalized single-cell sequencing has emerged as a way of identifying and characterizing the heterogeneity within cell populations, which is important for disease pathogenesis. In the future, single-cell circRNA sequencing may play a vital role in identifying different factors involved in interactions between tumor cells and stromal cells in the TME contributing to metastasis.

## Data Availability

Not applicable.

## Abbreviations

AR: Androgen receptor
BM: Basement membrane
CAFs: Cancer-associated fibroblasts
CC: Colon cancer
CCRCC: Clear cell renal cell carcinoma
CRC: Colorectal cancer
COL1A1: Collagen type I α1
CXCR4: C-X-C motif chemokine receptor 4
ECM: Extracellular matrix
EOC: Epithelial ovarian cancer
FN1: Fibronectin 1
GC: Gastric cancer
ISM: Isthmin
ITGB8: Integrin β8
HCC: Hepatocellular carcinoma
LUAD: Lung adenocarcinoma
MMP: Matrix metalloproteinase
MTDH: Metadherin
NFPAs: Non-functioning pituitary adenomas
NSCLC: Non-small cell lung cancer
OSCC: Oral squamous cell carcinoma
PAAD: Pancreatic adenocarcinoma
PDAC: Pancreatic ductal adenocarcinoma
SOX9: SRY (sex determining region Y)-box 9
TERT: Telomerase reverse transcriptase
TIMPs: tissue inhibitors of metalloproteinases
TME: Tumor microenvironment
TPT1: Translationally controlled tumor protein
VM: Vasculogenic mimicry
